# Polymorphisms in the airway epithelium related genes CDHR3 and EMSY are associated with asthma susceptibility

**DOI:** 10.1186/s12890-020-01334-0

**Published:** 2020-11-19

**Authors:** Miaomiao Zhang, Guo Chen, Yu Wang, Shou-Quan Wu, Andrew J. Sandford, Jian-Qing He

**Affiliations:** 1 Department of Respiratory and Critical Care Medicine, West China Hospital, Sichuan University, No. 37, Guo Xue Alley, Chengdu, Sichuan 610041 People’s Republic of China; 2 Department of Geriatrics, Sichuan Academy of Medical Sciences & Sichuan Provincial People’s Hospital, Chengdu, Sichuan China; 3 Chinese Academy of Sciences Sichuan Translational Medicine Research Hospital, Chengdu, Sichuan China; 4 Centre for Heart Lung Innovation, University of British Columbia and St. Paul’s Hospital, Vancouver, BC Canada

**Keywords:** *CDHR3*, *EMSY*, Asthma, Polymorphism, Susceptibility

## Abstract

**Background:**

As a main line of defense of the respiratory tract, the airway epithelium plays an important role in the pathogenesis of asthma. *CDHR3* and *EMSY* were reported to be expressed in the human airway epithelium. Although previous genome-wide association studies found that the two genes were associated with asthma susceptibility, similar observations have not been made in the Chinese Han population.

**Methods:**

A total of 300 asthma patients and 418 healthy controls unrelated Chinese Han individuals were enrolled. Tag-single nucleotide polymorphisms (Tag-SNPs) were genotyped and the associations between SNPs and asthma risk were analyzed by binary logistic regression analysis.

**Results:**

After adjusting for confounding factors, the A allele of rs3847076 in *CDHR3* was associated with increased susceptibility to asthma (OR = 1.407, 95% CI: 1.030–1.923). For the *EMSY* gene, the T alleles of both rs2508746 and rs12278256 were related with decreased susceptibility to asthma (additive model: OR = 0.718, 95% CI: 0.536–0.961; OR = 0.558, 95% CI: 0.332–0.937, respectively). In addition, the GG genotype of rs1892953 showed an association with increased asthma risk under the recessive model (OR = 1.667, 95% CI: 1.104–2.518) and the GATCTGAGT haplotype in *EMSY* was associated with reduced asthma risk (*P* = 0.037).

**Conclusions:**

This study identified novel associations of rs3847076 in *CDHR3*, as well as rs1892953, rs2508746 and rs12278256 in *EMSY* with adult asthma susceptibility in the Chinese Han population. Our observations suggest that *CDHR3* and *EMSY* may play important roles in the pathogenesis of asthma in Chinese individuals. Further study with larger sample size is needed.

**Supplementary Information:**

The online version contains supplementary material available at 10.1186/s12890-020-01334-0.

## Background

Asthma is a chronic airway inflammatory disease that affects populations throughout the world. A World Health Organization report [1] predicted that the number of asthma patients would increase to 400 million by 2025 and 250,000 patients may die from this disease each year. A recent survey indicated that the prevalence of asthma among individuals aged > 14 years was 1.24% and there are approximately 30 million asthmatic patients in China [2]. The pathogenesis of asthma is still incompletely understood but it is known that genetic factors play a significant part in asthma susceptibility. The heritability of asthma was estimated to be 60 to 70% in an Australian twin study [3]. Genetic factors contributed to 90% of the variance in the susceptibility to asthma in a 5-year-old twin pair study [4].

As the first barrier between the human body and the environment, the airway epithelium has an important role in regulating the inflammation, immunity and tissue repair in the pathogenesis of asthma [5]. One genome-wide association study (GWAS) of a Danish population identified Cadherin related family member 3 (*CDHR3*), which is highly expressed in human airway epithelium, as a susceptibility locus for childhood asthma with severe exacerbations [6]. A GWAS in 2017 demonstrated that Chromosome 11 open reading frame 30 *(C11orf30)*, also called *EMSY* or BRCA2-interacting transcriptional repressor, another gene expressed in airway epithelium [7], was a risk locus for food allergy in a Canadian population [8] and this gene has been shown to be involved in the epigenetic regulation of gene expression [9]. However, there have been few studies of these two genes in Chinese asthmatics. Therefore, this study aimed to investigate the association of common variants in *CDHR3* and *EMSY* with adult asthma in the Chinese population.

## Methods

### Study population

The inclusion and exclusion criteria of both healthy controls and asthma group was the same as previously described in our published article [10]. The asthmatic cases were diagnosed by at least three respiratory physicians from the West China Hospital. From September 2013 to September 2016, 3 ml of venous blood was collected from each unrelated subject and stored in a − 80 °C refrigerator. The study was approved by the ethical committee of the West China Hospital of Sichuan University (Protocol No. 23).

### Single Nucleotide Polymorphism (SNP) selection and genotyping

Tag-SNPs of *CDHR3* with minor allele frequency (MAF) ≥ 0.05 and r^2^ ≥ 0.64 were chosen as we performed before [10]. The final selected 23 tag-SNPs of *CDHR3* were rs3887998, rs12155008, rs41267, rs3892893, rs10270308, rs34426483, rs193795, rs2526978, rs381188, rs10241452, rs3847076, rs11981655, rs10808147, rs193806, rs2528883, rs41269, rs2526979, rs2526976, rs41262, rs41266, rs6967330, rs41270 and rs448024 (Table S1). The selection of SNPs in *EMSY* was the same as gene *CDHR3* except for r^2^ ≥ 0.80 and literature review [11–14]. The 17 SNPs of *EMSY* were rs3753051, rs7125744, rs7926009, rs4945087, rs2508740, rs1939469, rs7115331, rs1044265, rs12278256, rs2513513, rs2508755, rs2155219, rs2513525, rs2508746, rs1892953, rs7130588 and rs10899234 (Table S2). Genomic DNA was extracted as we performed previously [10]. As a quality control measure, 5% of randomly chosen samples, were repeated genotyped. Both genotype results reached concordance rate of 100%.

### Data analyses

Software Statistical Package for the Social Sciences (SPSS, SPSS Inc., Chicago, IL, USA), version 21.0, was used for statistical analyses, with *p* < 0.05 indicating statistically significant.

Genotype distributions under additive, dominant and recessive models were calculated by binary logistic regression analysis. Hardy-Weinberg equilibrium (HWE) among the controls was computed using plink software. Haploview and SHEsis software (http://analysis.bio-x.cn) were combined to perform linkage disequilibrium (LD) and haplotype analysis. Potential function of significant SNPs was predicted by the software RegulomeDB (http://www.regulomedb.org/) and Haploreg v4 (http://compbio.mit.edu/HaploReg). Three measures, RERI (relative excess risk due to interaction), AP (the attributable proportion due to interaction) and S (synergy index), were applied to calculate biological interactions [15]. RERI and AP equal 0 and S equals 1 means no biological interaction. The interaction between these significant SNPs and smoking (smoking status = 1,non-smoking status = 0), sex (male = 1,female = 0) and body mass index (BMI, BMI ≥ 24 = 1,BMI<24 = 0) was calculated.

## Results

### Subject characteristics

A total of 300 asthma patients and 418 healthy controls were enrolled. The average ages of asthma patients and controls were 43.6 ± 13.48 and 44.09 ± 13.75 years, respectively. No significant differences in sex, body mass index (BMI) and smoking history were observed between case and control groups (Table 1). Late-onset asthma (age of asthma onset ≥18 years) accounted for 74.3% in the case group. Most asthma individuals were outpatients (88.67%), and we could only get half of the patients’ reports of eosinophil count, total serum immunoglobulin E (IgE), pulmonary function test and provocation or relaxation test. The other half of the patients’ relevant tests were done in other hospitals, but we couldn’t acquire. 58.33% of the patients adopted the step 4 treatment plan according to Global Strategy for Asthma Management and Prevention (2018 update) [16], 12.67% adopted step 5, 3.33% used step 3 and the other patients’ treatment information was lost.

**Table 1:** Characteristics of cases and controls

Characteristic	Control(n%)	Case (n%)	*P* value
Gender			
Male	162 (38.76%)	118 (39.33%)	0.876
Female	256 (61.24%)	182 (60.67%)	
Age (mean ± SD,years)	44.09 ± 13.75	43.6 ± 13.48	0.64
Smoking status			
Current and ex-smokers	55 (13.16%)	49 (16.33%)	0.179
Non-smoking	207 (49.52%)	247 (82.33%)	
Smoking status unclear	156 (37.32%)	4 (1.33%)	
BMI (mean ± SD)	22.94 ± 3.34	23.11 ± 3.28	0.517
BMI < 24	227 (54.31%)	197 (65.67%)	
BMI ≥ 24	121 (29.67%)	103 (34.33%)	
Types of patients			
Emergency patients or inpatients		34 (11.33%)	
Outpatients		266 (88.67%)	
Asthma onset time			
Early-onset asthma(< 18 years old)		42 (14.00%)	
Late-onset asthma(≥18 years old)		223 (74.33%)	
Onset time unclear		35 (11.67%)	
Eosinophil count		171 (57.00%)	
Total IgE		139 (46.33%)	
Asthma with pulmonary function test		174 (58.00%)	
FEV1% predicted (mean ± SD)		83.61 ± 19.97	
FEV1/FVC%(mean ± SD)		72.37 ± 13.63	
Provocation test or relaxation test		153 (51%)	
Positive provocation test or relaxation test		134 (44.67%)	
Treatment scheme			
Step 3 treatment		10 (3.33%)	
Step 4 treatment		175 (58.33%)	
Step 5 treatment		38 (12.67%)	

Values are means ± standard deviation (SD) and absolute numbers (percentages). *BMI* body mass index; Early-onset asthma, age of asthma onset < 18 years; Late-onset asthma, age of asthma onset ≥18 years; FEV1, forced expiratory volume in 1 s; *FVC* forced vital capacity

### Association analyses between *CDHR3*, *EMSY* SNPs and asthma susceptibility

The characteristics of the selected SNPs are listed in Table S1 and S2. Rs10899234 in *EMSY* and rs6967330 in *CDHR3* were excluded due to their deviation from HWE in the control subjects (*P* < 0.05). The genotyping assays failed for rs12155008, rs41270 and rs448024 in *CDHR3*.

After adjusting for confounding factors including age, sex, BMI and smoking history, four SNPs were found to be associated with asthma susceptibility (Table 2 and Figure S1). The A allele of rs3847076 in *CDHR3* was associated with increased susceptibility to asthma under the additive model (*P* = 0.032, OR = 1.407, 95% CI: 1.030–1.923). For *EMSY*, both the TC/TT genotype and T allele of rs2508746 were associated with decreased risk of asthma (dominant model: *P* = 0.019, OR = 0.660, 95% CI: 0.465–0.935; additive model: *P* = 0.026, OR = 0.718, 95% CI: 0.536–0.961). The TG/TT genotype and T allele of rs12278256 were associated with reduced asthma risk (dominant model: *P* = 0.033, OR = 0.563, 95% CI: 0.332–0.953; additive model: *P* = 0.027, OR = 0.558, 95% CI: 0.332–0.937). Finally, the GG genotype of rs1892953 showed an association with increased asthma risk under the recessive model (*P* = 0.015, OR = 1.667, 95% CI: 1.104–2.518). After excluding people who were lack of smoking or BMI information, we used the online software SNPStats (https://snpstats.net/) for statistical analysis again and the results (shown in the Table S3) were similar to Table 1. However, it should be reminded that some significant associations maybe were expected just by chance.

**Table 2:** The four SNPs associated with asthma

Genes	SNPs	Genetic models	Genotypes	Control n(%)	Case n(%)	P*	OR 95%CI^*^
*CDHR3*	rs3847076	Dom	CC	285 (68.2)	185 (61.7)	0.081	1.378 (0.962–1.973)
			CA + AA	133 (31.8)	115 (38.3)		
		Rec	CC + CA	408 (97.6)	285 (95.0)	0.060	2.689 (0.958–7.545)
			AA	10 (2.4)	15 (5.0)		
		Add	CC/CA/AA			0.032*	1.407 (1.030–1.923)*
*EMSY*	rs2508746	Dom	CC	244 (58.4)	197 (65.7)	0.019*	0.660 (0.465–0.935)*
			TC + TT	174 (41.6)	103 (34.3)		
		Rec	CC + TC	396 (94.7)	288 (96.0)	0.445	0.733 (0.331–1.626)
			TT	22 (5.3)	12 (4.0)		
		Add	CC/TC/TT			0.026*	0.718 (0.536–0.961)*
*EMSY*	rs1892953	Dom	AA	115 (27.5)	76 (25.3)	0.647	1.094 (0.745–1.605)
			GA + GG	303 (72.5)	224 (74.7)		
		Rec	AA+GA	319 (76.3)	219 (73.0)	0.015*	1.667 (1.104–2.518)*
			GG	99 (23.7)	81 (27.0)		
		Add	AA/GA/GG			0.081	1.240 (0.974–1.579)
*EMSY*	rs12278256	Dom	GG	357 (85.4)	272 (90.7)	0.033*	0.563 (0.332–0.953)*
			TG + TT	61 (14.6)	28 (9.3)		
		Rec	GG + TG	417 (99.8)	300 (100)	1	–
			TT	1 (0.2)	0 (0)		
		Add	GG/TG/TT			0.027*	0.558 (0.332–0.937)*

*Adjusted for sex, age, body mass index and smoking history with logistic regression, *P* < 0.05

*Add* additive model, *Dom* dominant model, *Rec* recessive model

Stratified analysis results by gender, smoking status, BMI status and onset age of asthma were shown in Table 3. The cut-off point of adult BMI in China is different from other countries, as 18.5 ≤ BMI < 24 kg/m^2^ meaning normal weight range and BMI ≥ 24 kg/m^2^ meaning overweight or obese [17]. Allele A of rs3847076 was associated with increased susceptibility to asthma in male subgroup, smoking subgroup, BMI < 24 kg/m^2^ subgroup and late onset asthma subgroup (*P* = 0.023, OR = 1.869; *P* = 0.009, OR = 2.168; *P* = 0.005, OR = 1.835 and *P* = 0.023, OR = 1.457, respectively). Similarly, rs2508746 TC + TT was related with decreased asthma susceptibility in the non-smoking subgroup, non-overweight subgroup, and late-onset asthma subgroup in dominant model (*P* = 0.014, OR = 0.618; *P* = 0.027, OR = 0.612 and *P* = 0.016, OR = 0.637, respectively). Meanwhile, rs1892953 GG shown increased risk of asthma in the female subgroup, non-smoking subgroup, non-overweight subgroup, and late onset asthma subgroup in recessive model (*P* = 0.038, OR = 1.738; *P* = 0.04, OR = 1.615; *P* = 0.017, OR = 1.910 and *P* = 0.017, OR = 1.680, respectively). Rs12278256 T was still associated with decreased asthma susceptibility in female subgroups, non-smoking subgroups, and non-overweight subgroups in additive model (*P* = 0.032, OR = 0.465; *P* = 0.02, OR = 0.508 and *P* = 0.028, OR = 0.481, respectively). The interaction between these four SNPs and smoking, sex and BMI were shown in Table S4. We got significant interaction between rs3847076 and rs1892953 and smoking, sex and BMI, while no interaction was found between rs12278256 and these clinical phenotypes. Meanwhile, significant interaction could also be observed between rs2508746 and either gender or BMI.

**Table 3:** Results of stratification analysis based on gender, smoking status, BMI status, and onset age of asthma

SNPs	Genetic models		Stratified by gender			Stratified by smoking status			Stratified by BMI status			Stratified by onset age of asthma	
			*P*	OR 95%CI		*P*	OR 95%CI		*P*	OR 95%CI		*P*	OR 95%CI
rs3847076	Dom	male	0.048*	**1.834 (1.005–3.347)**	smoking	0.018*	**2.252 (1.149–4.413)**	BMI<24	0.004*	**1.925 (1.233–3.007)**	late onset asthma	0.063	1.428 (0.981–2.077)
	Rec		0.115	5.656 (0.654–48.882)		0.09	4.222 (0.799–22.320)		0.12	2.835 (0.761–10.559)		0.049*	**2.861 (1.006–8.134)**
	Add		0.023*	**1.869 (1.091–3.202)**		0.009*	**2.168 (1.212–3.872)**		0.005*	**1.835 (1.234–2.726)**		0.023*	**1.457 (1.054–2.013)**
rs2508746	Dom	female	–	–	non-smoking	0.014*	**0.618 (0.420–0.908)**	BMI<24	0.027*	**0.612 (0.396–0.946)**	late onset asthma	0.016*	**0.637 (0.441–0.919)**
	Rec		–	–		0.498	0.737 (0.304–1.782)		0.862	0.920 (0.361–2.347)		0.419	0.706 (0.304–1.641)
	Add		–	–		0.022*	**0.685 (0.495–0.947)**		0.06	0.710 (0.496–1.015)		0.021*	**0.696 (0.511–0.948)**
rs1892953	Dom	female	0.548	1.159 (0.717–1.873)	non-smoking	0.456	0.174 (0.770–1.790)	BMI<24	0.702	1.097 (0.682–1.766)	late onset asthma	0.692	1.084 (0.726–1.620)
	Rec		0.038*	**1.738 (1.031–2.927)**		0.04*	**1.615 (1.021–2.553)**		0.017*	**1.910 (1.123–3.250)**		0.017*	**1.680 (1.095–2.578)**
	Add		0.108	1.282 (0.947–1.737)		0.091	1.259 (0.964–1.644)		0.096	1.297 (0.955–1.761)		0.094	1.241 (0.964–1.599)
rs12278256	Dom	female	0.037*	**0.468 (0.229–0.955)**	non-smoking	0.023*	**0.512 (0.287–0.913)**	BMI<24	0.033*	**0.485 (0.249–0.944)**	late onset asthma	–	–
	Rec		1			1			1			–	–
	Add		0.032*	**0.465 (0.231–0.936)**		0.02*	**0.508 (0.288–0.897)**		0.028*	**0.481 (0.250–0.923)**		–	–

*Adjusted for sex, age, body mass index and smoking history with logistic regression, *P* < 0.05

*Add* additive model, *Dom* dominant model, *Rec* recessive model

We further explored the relationship between eosinophil count, total serum IgE, pulmonary function test of asthma patients and gene variants. Eosinophil count was higher in asthma patients with genotype CC of rs3847076 comparing to individuals with genotype CA (Table S5). Total IgE was related with four variants of *CDHR3* and one variant of *EMSY* (Table S6). Both FEV1% predicted and FEV1/FVC% were significant different in nine SNP genotypes, including rs2508746 and rs1892953. Higher FEV1/FVC% was also seen in genotype GG of rs12278256 (Table S7). Due to the small number of samples, further verification research is needed.

### Haplotype and LD analysis

**Fig. 1:**
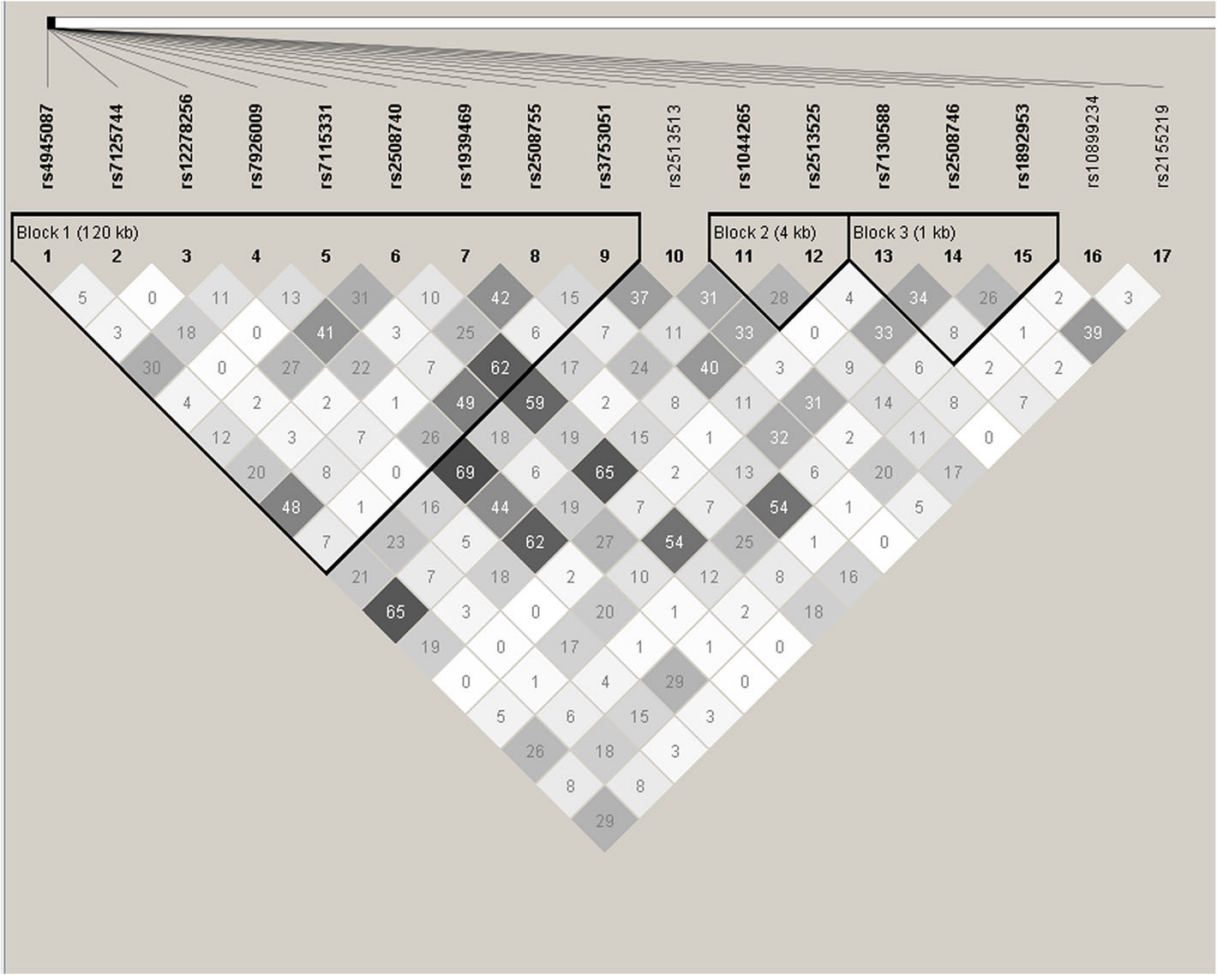
the Analysis of linkage disequilibrium of 17 SNPs in *EMSY.* Note: Each square represents the linkage disequilibrium of two corresponding SNPs, which is displayed as r^2^ × 100. The larger the darkness of the square, the larger the value of r^2^ × 100

**Fig. 2:**
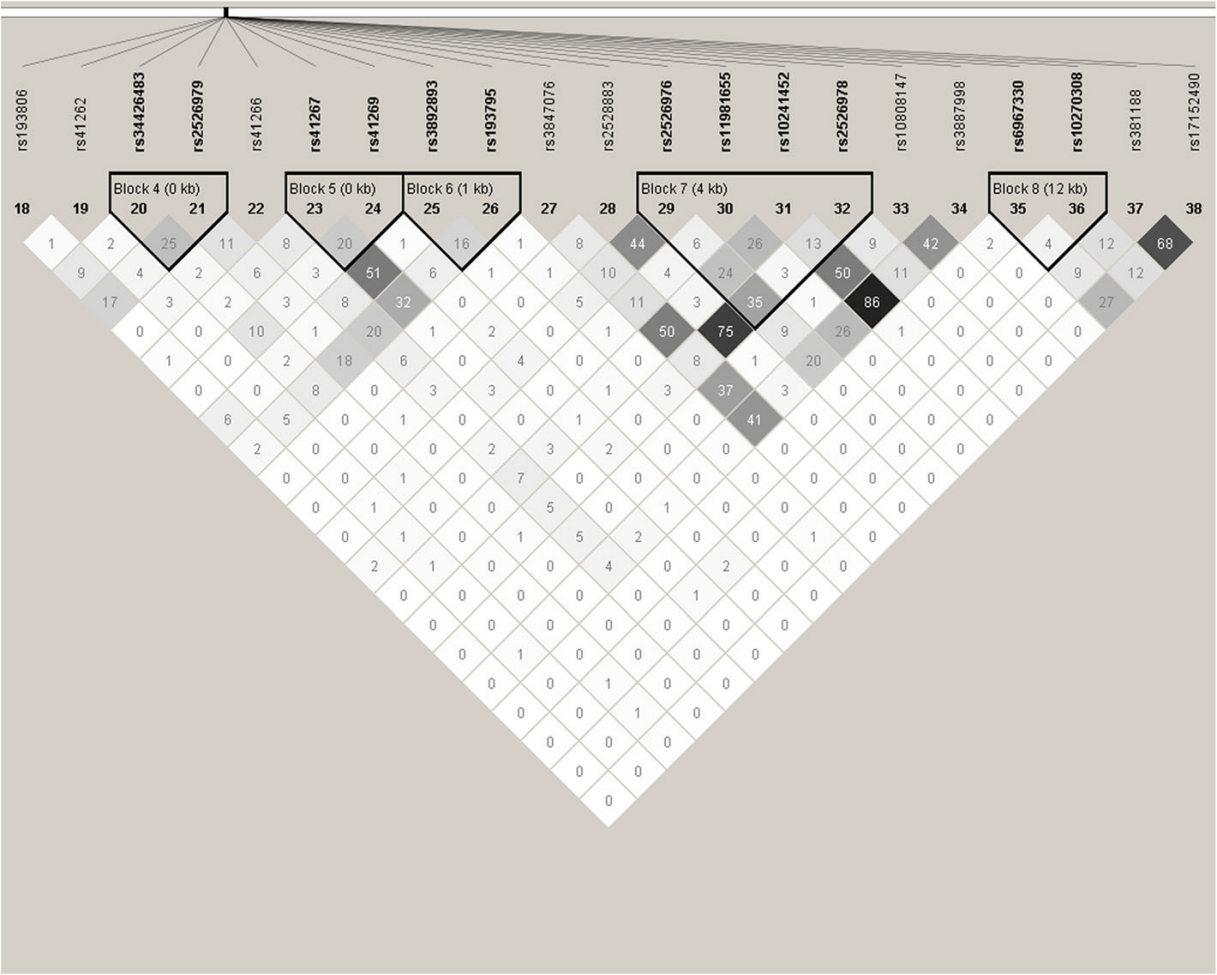
the Analysis of linkage disequilibrium of 20 tag-SNPs in *CDHR3.* Note: Each square represents the linkage disequilibrium of two corresponding SNPs, which is displayed as r^2^ × 100. The larger the darkness of the square, the larger the value of r^2^ × 100

The LD between SNPs of *CDHR3* and *EMSY* was low and those SNPs were divided into eight haplotype blocks with Haploview software (Figs. 1 and 2). Only the haplotype consisting of GATCTGAGT in block 1 of *EMSY* was associated with decreased risk of asthma (*P* = 0.037, OR = 0.615, 95% CI: 0.388–0.975) (Table 4).

**Table 4:** The association between *EMSY* haplotypes in block 1 and asthma susceptibility

Haplotype	Case N (%)	Control N (%)	Chi^2^	Pearson’s p	OR (95% CI)
AAGTTAAAT	207.00 (0.345)	285.58 (0.342)	0	0.999	1.000 (0.801–1.248)
GAGCGGAGC	43.00 (0.072)	57.59 (0.069)	**0.023**	**0.878**	**1.033 (0.685–1.556)**
GAGCTAAAT	44.00 (0.073)	58.04 (0.069)	0.054	0.815	1.050 (0.699–1.577)
GAGCTGAGC	35.00 (0.058)	52.18 (0.062)	0.134	0.714	0.921 (0.592–1.432)
GAGTTAGGT	178.00 (0.297)	230.22 (0.275)	0.589	0.443	1.095 (0.868–1.382)
GATCTGAGT	28.00 (0.047)	61.00 (0.073)	4.346	0.037*	0.615 (0.388–0.975)*
GGGCTAAAT	60.00 (0.100)	76.32 (0.091)	0.246	0.62	1.094 (0.766–1.562)
Global result	600	836	4.912565	0.555	

For each haplotype, alleles were arranged in order of rs4945087, rs7125744, rs12278256, rs7926009, rs7115331, rs2508740, rs1939469, rs2508755 rs3753051. * means two-sided *P*<0.05

### Functional prediction results

Four statistically significant SNPs were predicted using the software RegulomeDB and Haploreg v4 (Table S8). Rs144934374 is strongly linked to rs12278256 and its RegulomeDB scores is lower than that of rs12278256, suggesting that it may be the functional site represented by rs12278256. Acting as promoter histone marks or enhancer histone marks, or affecting DNAse is suggested to be associated with chromatin status, and binding proteins or altering regulatory motifs in ChIP-Seq suggest that transcription levels may be affected. It seems that these four SNPs may have certain effects on chromatin status and transcription level. Rs1892953 appears as an expression quantitative trait loci (eQTL) SNP in thyroid tissue [13].

## Discussion

In this group of Chinese Han adults, the relationship between two airway epithelial-related genes *EMSY* and *CDHR3* and risk of asthma were investigated, and four polymorphisms related to asthma susceptibility were obtained, which were rs3847076 of *CDHR3* and rs2508746, rs1892953 and rs12278256 of *EMSY*. A further subgroup analysis of these four variants revealed that their association with asthma was present in different subgroups.

*CDHR3*, located on chromosome 7, is specifically expressed in ciliated airway epithelial cells which are the targets of Rhinovirus C (RV-C) infection, and its expression was positively associated with RV-C binding, replication and entry into the host cells [18, 19]. There are only a few studies describing the relationship between *CDHR3* polymorphisms and asthma, and the results were inconsistent in different populations. The A allele of rs6967330 in *CDHR3* increased the risk of wheezing and hospitalizations for childhood asthma in a Danish study [6]. Rs17152490, in LD with rs6967330, was reported to affect asthma risk through *cis*-regulation of its gene expression in human bronchial epithelial cells [20] . However, rs6967330 was only related to early-onset asthma in a Japanese population [21] and no association between rs6967330 and asthma was found in Chinese children [22]. In the present study, rs6967330 was not in HWE and our data suggest that rs3847076 may increase the risk of asthma in adults, which were inconsistent with the previous studies. The potential reasons for this discrepancy are as follows: Firstly, the susceptibility to asthma may differ in different populations, and secondly, late-onset asthma patients accounted for the majority of the case group in this study, in contrast to the above Japanese study which reported the positive relationship between rs6967330 and early-onset asthma in children. A future study of different asthma phenotypes would be beneficial to the accurate prevention and treatment of asthma.

Peripheral blood eosinophil was one of the main inflammatory cells involved in asthma and other allergic diseases [23]. Meta-analysis showed that the level of eosinophil in peripheral blood could better reflect the inflammatory status of eosinophil in airway [24], predicted the trend of long-term decline of lung function [25] and the risk of asthma attack in adults and children [26]. And more research is needed to determine whether rs3847076 genotypes of *CDHR3* are related to the number of eosinophils. Some studies have shown that the serum total IgE level was related to the severity and control of asthma [27]. The relationship between *CDHR3* variants and total IgE needed further investigated.

*EMSY*, located on chromosome 11q13.5, is expressed in the human airway epithelium and encoded by the EMSY protein. GWAS studies showed that *EMSY* was involved in allergic diseases including atopic dermatitis and food allergy [28, 29]. Several SNPs, rs7130588, rs10899234, rs6592657, as well as SNPs rs2508746 and rs1892953 that we studied were associated with total serum IgE levels in non-Hispanic Caucasian asthmatic patients [11]. In an eQTL analysis, Li et al. [20] reported that rs2508740, rs2513525, rs4300410 (in complete LD with rs7926009), rs10793169 (in complete LD with rs7926009), rs2513513 and rs4245443 were significantly correlated with mRNA expression levels of *EMSY* in human bronchial alveolar lavage. Another GWAS study reported that rs7130588 in *EMSY* was associated with asthma [30]. A meta-analysis demonstrated that rs2155219 in *EMSY* increased the risk of allergic sensitization [12]. In the present study, three SNPs (rs2508746, rs1892953 and rs12278256) were related to asthma susceptibility in the Chinese Han population, of which rs12278256 has not been reported in previous studies. As a variant located in the upstream region of *EMSY,* rs12278256 might affect the regulatory motifs and chromatin status of this gene and further study is needed to verify this hypothesis. Based on our results, rs2508746, rs1892953 and rs12278256 genotypes were associated with level of FEV1% predicted and/or FEV1/FVC%, which also suggested that gene EMSY was likely related with lung function.

Studies in the twin population have shown that susceptibility to asthma can be attributed to genetic factors [3, 4]. Although current genome-wide association studies have identified numerous polymorphisms associated with asthma susceptibility, the odds ratio (OR) is around 1.2, and only a small percentage of asthma prevalence can be contributed to them. Some experts have proposed to study the interaction between genes and environment [31, 32]. It is well known that environmental factors such as smoking and obesity are susceptibility factors for asthma, but the specific mechanism is not clear. A number of studies have shown that smoking is associated with increased risk of asthma, reduced efficacy of inhaled corticosteroids treatment, acute exacerbations, and airway remodeling in asthma [33–37]. Mechanisms of asthma in the obese may include mechanical factors and inflammatory immunity [38]. Studies have shown that the SNPs at 17q21.2 is associated with BMI levels in asthmatic patients [39]. Functional prediction suggests that the alternate A allele of rs3847076 decrease the effect on motif TCF4 relative to the reference C allele, according to the library [40].

Recently, genetic studies have detected a lot of susceptibility genes for asthma. This study was the first attempt to investigate the association between *CDHR3*, *EMSY* and adult asthma susceptibility in the Chinese Han population. We found rs3847076 in *CDHR3*, rs2508746, rs1892953 and rs12278256 in *EMSY* were associated with the risk of adult asthma. However, there were some limitations to this study. Adjustment was not performed to correct the results for multiple testing, due to the weak effect of each single polymorphism on asthma susceptibility. In addition, the allergic phenotypes of the asthma patients were not clear and serum IgE levels were not analyzed in the study. Lastly, *CDHR3* is a huge gene spanning over 159 kb and the strategy of tag-SNPs selection with r^2^ > 0.64 in this study may have missed some SNPs associated with the disease.

## Conclusions

In conclusion, this study is the first to identify that the airway epithelium related genes *EMSY* and *CDHR3* were associated with adult asthma susceptibility in the Chinese Han population. The *CDHR3* rs3847076 allele A and *EMSY* rs1892953 genotype GG may increase the risk of asthma. The *EMSY* rs2508746 and rs12278256 allele T may decrease asthma risk. A population with a larger sample size is needed for further exploration of the association.

## Supplementary Information

**Additional file 1:** **Table S1.** Characteristics of Tag-SNPs in *CDHR3.* **Table S2.** Characteristics of the SNPs in *EMSY.* **Table S3.** The four SNPs associated with asthma susceptibility in remaining 549 individuals after excluding subjects with missing information on smoking or BMI. **Table S4.** Interaction between four SNPs and smoking, gender and BMI. **Table S5.** The two SNPs associated with the number of Eosinophil cell. **Table S6.** The five SNPs associated with total IgE. **Table S7.** The 13 SNPs associated with FEV1% predicted and 14 SNPs associated with FEV1/FVC%. **Table S8.** Functional prediction results by softwares RegulomeDB and HaploReg v4. **Figure S1.** The volcano plots of significant SNPs.

**Additional file 2.**

## Data Availability

All data of the study are available in the excel named “Additional file 2”.

## Abbreviations

Tag-SNPs: Tag-single nucleotide polymorphisms
GWAS: Genome-Wide Association Study
*CDHR3*: Cadherin related family member 3
*C11orf30*: Chromosome 11 open reading frame 30
SNPs: Single Nucleotide Polymorphisms
MAF: Minor Allele Frequency
PCR: Polymerase Chain Reaction
SPSS: Statistical Package for the Social Sciences
HWE: Hardy-Weinberg equilibrium
LD: Linkage Disequilibrium
BMI: Body Mass Index
RV-C: Rhinovirus C
IgE: Immunoglobulin E
eQTL: Expression Quantitative Trait Loci
OR: Odds Ratio
