# Supplementary material for: Polymorphisms in the airway epithelium related genes CDHR3 and EMSY are associated with asthma susceptibility
Source: BMC Pulm Med. 2020 Nov 19;20:305. doi: 10.1186/s12890-020-01334-0 (PMC7678090; doi:10.1186/s12890-020-01334-0)
Supplement: Supplementary file 1 — Additional file 1: Table S1. Characteristics of Tag-SNPs in CDHR3. Table S2. Characteristics of the SNPs in EMSY. Table S3. The four SNPs associated with asthma susceptibility in remaining 549 individuals after excluding subjects with missing information on smoking or BMI. Table S4. Interaction between four SNPs and smoking, gender and BMI. Table S5. The two SNPs associated with the number of Eosinophil cell. Table S6. The five SNPs associated with total IgE. Table S7. The 13 SNPs associated with FEV1% predicted and 14 SNPs associated with FEV1/FVC%. Table S8. Functional prediction results by softwares RegulomeDB and HaploReg v4. Figure S1. The volcano plots of significant SNPs. [file 12890_2020_1334_MOESM1_ESM.doc]

**Supplementary Materials**

Table S1 Characteristics of Tag-SNPs in *CDHR3*

| chromosome | SNPs | SNPs location | Function | Minor allele | MAF* | MAF | HWE^#^  *P* value | source |
| --- | --- | --- | --- | --- | --- | --- | --- | --- |
| 7 | rs3892893 | 105979536 | intron variant | C | 0.13 | 0.14 | 0.17 | Tag-SNP |
| 7 | rs10270308 | 106030821 | synonymous variant | C | 0.39 | 0.39 | 1 | Tag-SNP |
| 7 | rs34426483 | 105974980 | missense variant | C | 0.24 | 0.28 | 0.18 | Tag-SNP |
| 7 | rs193795 | 105981066 | synonymous variant | G | 0.47 | 0.5 | 0.49 | Tag-SNP |
| 7 | rs2526978 | 105999714 | intron variant | T | 0.35 | 0.3 | 0.82 | Tag-SNP |
| 7 | rs381188 | 106034144 | 3 prime UTR variant | C | 0.14 | 0.17 | 0.49 | Tag-SNP |
| 7 | rs10241452 | 105997129 | intron variant | T | 0.21 | 0.22 | 0.32 | Tag-SNP |
| 7 | rs3847076 | 105989994 | intron variant | A | 0.17 | 0.17 | 0.6 | Tag-SNP |
| 7 | rs11981655 | 105997086 | intron variant | G | 0.08 | 0.08 | 0.5 | Tag-SNP |
| 7 | rs10808147 | 106000090 | intron variant | T | 0.16 | 0.16 | 1 | Tag-SNP |
| 7 | rs193806 | 105963785 | intron variant | C | 0.19 | 0.21 | 0.66 | Tag-SNP |
| 7 | rs2528883 | 105993995 | intron variant | A | 0.42 | 0.35 | 0.45 | Tag-SNP |
| 7 | rs41269 | 105979351 | intron variant | A | 0.07 | 0.07 | 0.46 | Tag-SNP |
| 7 | rs2526979 | 105975969 | intron variant | A | 0.47 | 0.39 | 0.61 | Tag-SNP |
| 7 | rs2526976 | 105995594 | intron variant | A | 0.38 | 0.47 | 0.69 | Tag-SNP |
| 7 | rs41262 | 105974101 | intron variant | T | 0.05 | 0.06 | 1 | Tag-SNP |
| 7 | rs41266 | 105978723 | intron variant | G | 0.3 | 0.34 | 0.44 | Tag-SNP |
| 7 | rs6967330 | 106018005 | missense variant | A | 0.05 | 0.07 | 0.018^#^ | Tag-SNP |
| 7 | rs3887998 | 106013421 | intron variant | A | 0.21 | 0.22 | 0.47 | Tag-SNP |
| 7 | rs12155008^&^ | 105995848 | intron variant | A | 0.11 | - | - | Tag-SNP |
| 7 | rs41267 | 105978760 | intron variant | C | 0.24 | 0.25 | 0.51 | Tag-SNP |
| 7 | rs41270^&^ | 105980583 | intron variant | A | 0.10 | - | - | Tag-SNP |
| 7 | rs448024^&^ | 106020052 | intron variant | T | 0.10 | - | - | Tag-SNP |

*Han Chinese in Beijing, China, from the 1000genomes database. HWE^#^, Hardy-Weinberg equilibrium in control group. &, The SNP failed to genotype. UTR, untranslated region. MAF, minor allele frequency in our experience.

*Table S2 Characteristics of the SNPs in EMSY*

| chromosome | SNPs | Function | Minor allele | MAF^*^ | MAF | HWE^#^  *P* value | | source |
| --- | --- | --- | --- | --- | --- | --- | --- | --- |
| 11 | rs3753051 | synonymous variant | C | 0.15 | 0.14 | 1 | Tag-SNP | |
| 11 | rs7125744 | 5'-flanking | G | 0.09 | 0.1 | 1 | Tag-SNP | |
| 11 | rs7130588 | 3'-flanking | G | 0.09 | 0.1 | 1 | literature | |
| 11 | rs10899234 | 3'-flanking | A | 0.18 | 0.19 | 0.036^#^ | literature | |
| 11 | rs2508740 | intron variant | G | 0.2 | 0.21 | 0.56 | Tag-SNP | |
| 11 | rs1939469 | intron variant | G | 0.33 | 0.28 | 0.34 | Tag-SNP | |
| 11 | rs7115331 | intron variant | G | 0.06 | 0.07 | 0.71 | Tag-SNP | |
| 11 | rs1044265 | 3 prime UTR variant | G | 0.42 | 0.44 | 0.77 | Tag-SNP | |
| 11 | rs2155219 | 3'-flanking | G | 0.41 | 0.45 | 0.84 | literature | |
| 11 | rs7926009 | intron variant | C | 0.35 | 0.38 | 0.47 | Tag-SNP | |
| 11 | rs2513525 | 3'-flanking | T | 0.27 | 0.28 | 0.12 | literature | |
| 11 | rs4945087 | 5'-flanking | A | 0.33 | 0.35 | 0.59 | Tag-SNP | |
| 11 | rs2508746 | 3'-flanking | T | 0.24 | 0.23 | 0.89 | literature | |
| 11 | rs1892953 | 3'-flanking | G | 0.44 | 0.48 | 0.7 | literature | |
| 11 | rs12278256 | upstream variant 2KB | T | 0.06 | 0.07 | 0.72 | Tag-SNP | |
| 11 | rs2513513 | 3 prime UTR variant | A | 0.29 | 0.31 | 0.91 | Tag-SNP | |
| 11 | rs2508755 | intron variant | A | 0.47 | 0.51 | 1 | Tag-SNP | |

*Han Chinese in Beijing, China, from the 1000genomes database. HWE^#^, Hardy-Weinberg equilibrium in control group. UTR, untranslated region. MAF, minor allele frequency in our experience.

Table S3 The four SNPs associated with asthma susceptibility in remaining 549 individuals after excluding subjects with missing information on smoking or BMI.

| Genes | SNPs | Genetic models | Genotypes | P | OR 95%CI |
| --- | --- | --- | --- | --- | --- |
| *CDHR3* | rs3847076 | Dominant | C/C | 0.085 | 1.37(0.96-1.97) |
|  |  |  | C/A-A/A |  |  |
|  |  | Recessive | C/C-C/A | 0.046 | 2.69(0.96-7.54) |
|  |  |  | A/A |  |  |
|  |  | additive | CC/CA/AA | **0.032*** | **1.40(1.03-1.92)*** |
| *EMSY* | rs2508746 | Dominant | C/C | **0.02*** | **0.66(0.47-0.94)*** |
|  |  |  | T/C-T/T |  |  |
|  |  | Recessive | C/C-T/C | 0.45 | 0.73(0.33-1.63) |
|  |  |  | T/T |  |  |
|  |  | additive | CC/TC/TT | **0.026*** | **0.72(0.54-0.96)*** |
| *EMSY* | rs1892953 | Dominant | A/A | 0.67 | 1.09(0.74-1.60) |
|  |  |  | G/A-G/G |  |  |
|  |  | Recessive | A/A-G/A | **0.013*** | **1.68(1.11-2.54)*** |
|  |  |  | G/G |  |  |
|  |  | additive | AA/GA/GG | 0.081 | 1.24(0.97-1.58) |
| *EMSY* | rs12278256 | Dominant | G/G | **0.032*** | **0.56(0.33-0.96)*** |
|  |  |  | T/G-T/T |  |  |
|  |  | Recessive | G/G-T/G | 0.24 | 0.00(0.00-NA) |
|  |  |  | T/T |  |  |
|  |  | additive | GG/TG/TT | **0.026*** | **0.56(0.33-0.94)*** |

* Adjusted for sex, age, body mass index and smoking history with logistic regression, P<0.05.

Table S4 Interaction between four SNPs and smoking, gender and BMI

| SNPs | Genetic models | Measure | smoking | gender | BMI |
| --- | --- | --- | --- | --- | --- |
|  |  |  | Estimate 95%CI | Estimate 95%CI | Estimate 95%CI |
| rs3847076＆smoking/gender/BMI | Dominant model | RERI | **12.16(0.58~23.75)** | **66.78(24.6~108.96)** | **53.69(17.34~90.03)** |
|  |  | AP | **0.72(0.52~0.92)** | **0.92(0.87~0.96)** | **0.82(0.72~0.92)** |
|  |  | S | **4.44(1.95~10.12)** | **15.74(8.5~29.13)** | **6.18(3.32~11.51)** |
|  | Recessive model | RERI | 141.85(-196.27~479.98) | 47515.11(-55958.41~150988.65) | 129.06(-84.88~343.02) |
|  |  | AP | 0.93(0.78~1.09) | 0.99(0.99~1.00) | 0.88(0.65~1.1) |
|  |  | S | 17.87(1.41~226.21) | 5690.98(539.97~59979.34) | 8.85(1.28~61.07) |
| rs2508746＆smoking/gender/BMI | Dominant model | RERI | 2.21(-1.28~5.7) | **14.08(4.39~23.78)** | **4.26(0.64~7.88)** |
|  |  | AP | 0.46(0.03~0.88) | **0.73(0.58~0.87)** | **0.57(0.34~0.81)** |
|  |  | S | 2.41(0.8~7.25) | **4.36(2.38~7.97)** | **3.02(1.43~6.36)** |
|  | Recessive model | RERI | 2.9(-8.41~14.22) | 15.96(-10.18~42.11) | 3.61(-8.53~15.75) |
|  |  | AP | 0.47(-0.54~1.49) | 0.78(0.49~1.07) | 0.49(-0.39~1.37) |
|  |  | S | 2.3(0.21~24.99) | 5.69(1.31~24.61) | 2.31(0.29~18.17) |
| rs1892953＆smoking/gender/BMI | Dominant model | RERI | **8.4(0.97~15.83)** | **52.71(21.38~84.05)** | **20.07(8.55~31.58)** |
|  |  | AP | **0.66(0.44~0.87)** | **0.88(0.83~0.94)** | **0.79(0.69~0.88)** |
|  |  | S | **3.54(1.64~7.63)** | **10.4(5.89~18.38)** | **5.71(3.32~9.80)** |
|  | Recessive model | RERI | 24.78(-4.02~53.59) | **200.47(54.85~346.1)** | **46.11(13.17~79.05)** |
|  |  | AP | 0.8(0.6~0.99) | **0.96(0.93~0.98)** | **0.84(0.74~0.95)** |
|  |  | S | 5.77(2.05~16.27) | **29.5(14.22~61.19)** | **7.34(3.46~15.60)** |
| rs12278256＆smoking/gender/BMI | Dominant model | RERI | 1.99(-3.63~7.62) | 9.7(-0.55~19.96) | 3.21(-1.76~8.20) |
|  |  | AP | 0.45(-0.27~1.18) | 0.68(0.44~0.93) | 0.52(0.10~0.95) |
|  |  | S | 2.41(0.39~14.76) | 3.85(1.57~9.47) | 2.73(0.84~8.82) |
|  | Recessive model | - | - | - | - |

Notes: 95%CI, 95% confidence interval; RERI, relative excess risk due to interaction; AP, attributable proportion due to interaction; S, synergy index. The 95% CI of RERI and AP contains 0, and the 95% CI of S contains 1 indicating no additive interaction.

Table S5 The two SNPs associated with the number of Eosinophil cell.

| Gene | SNP | Genotypes | number | Mean eosinophil cell (10^9/L) | P |
| --- | --- | --- | --- | --- | --- |
| CDHR3 | rs3847076 | CC | 108 | 0.3234 |  |
|  |  | AA+CA | 63 | 0.2413 | 0.093 |
|  |  | CC+CA | 164 | 0.2931 |  |
|  |  | AA | 7 | 0.2943 | 0.992 |
|  |  | CC | 108 | 0.3234 |  |
|  |  | CA | 56 | 0.2346 | **0.044** |
|  |  | CC | 108 | 0.3234 |  |
|  |  | AA | 7 | 0.2943 | 0.827 |
| CDHR3 | rs10808147 | CC | 110 | 0.3049 |  |
|  |  | TT+TC | 61 | 0.272 | 0.505 |
|  |  | CC+TC | 164 | 0.3015 |  |
|  |  | TT | 7 | 0.0986 | **<0.001** |
|  |  | CC | 110 | 0.3049 |  |
|  |  | TC | 54 | 0.2944 | 0.841 |
|  |  | CC | 110 | 0.3049 |  |
|  |  | TT | 7 | 0.0986 | **<0.001** |

Table S6 The five SNPs associated with total IgE.

| Gene | SNP | genotype | number | Mean total IgE (IU/ml) | P |
| --- | --- | --- | --- | --- | --- |
| CDHR3 | rs41267 | TT | 78 | 264.59 |  |
|  |  | CC+TC | 61 | 153.23 | **0.04** |
|  |  | TT+TC | 132 | 215.68 |  |
|  |  | CC | 7 | 216.44 | 0.995 |
|  |  | TT | 78 | 264.59 |  |
|  |  | GC | 54 | 145.03 | **0.026** |
|  |  | TT | 78 | 264.59 |  |
|  |  | CC | 7 | 216.44 | 0.765 |
| CDHR3 | rs2526978 | CC | 65 | 149.90 |  |
|  |  | TT+TC | 74 | 273.53 | **0.026** |
|  |  | CC+TC | 130 | 218.75 |  |
|  |  | TT | 9 | 171.91 | 0.691 |
|  |  | CC | 65 | 149.90 |  |
|  |  | TC | 65 | 287.60 | **0.025** |
|  |  | CC | 65 | 149.90 |  |
|  |  | TT | 9 | 171.91 | 0.738 |
| CDHR3 | rs11981655 | AA | 121 | 230.07 |  |
|  |  | GG+GA | 18 | 119.21 | **0.014** |
|  |  | AA+GA | 137 | 217.36 |  |
|  |  | GG | 2 | 103.15 | 0.639 |
|  |  | AA | 121 | 230.07 |  |
|  |  | GA | 16 | 121.22 | **0.023** |
|  |  | AA | 121 | 230.07 |  |
|  |  | GG | 2 | 103.15 | 0.62 |
| CDHR3 | rs2528883 | GG | 63 | 154.57 |  |
|  |  | AA+GA | 76 | 266.41 | **0.041** |
|  |  | GG+GA | 124 | 221.42 |  |
|  |  | AA | 15 | 168.58 | 0.572 |
|  |  | GG | 63 | 154.57 |  |
|  |  | GA | 61 | 290.47 | **0.036** |
|  |  | GG | 63 | 154.57 |  |
|  |  | AA | 15 | 168.57 | 0.793 |
| EMSY | rs4945087 | GG | 62 | 229.57 |  |
|  |  | AA+GA | 77 | 204.56 | 0.668 |
|  |  | GG+GA | 121 | 233.36 |  |
|  |  | AA | 18 | 97.12 | **0.001** |
|  |  | GG | 62 | 229.57 |  |
|  |  | GA | 59 | 237.34 | 0.906 |
|  |  | GG | 62 | 229.57 |  |
|  |  | AA | 18 | 97.12 | **0.008** |

Table S7 The thirteen SNPs associated with FEV1 % predicted and fourteen SNPs associated with FEV1/FVC%.

| Gene | SNP | Genotype | Number | Mean FEV1 % predicted | P | Mean FEV1/FVC% | P |
| --- | --- | --- | --- | --- | --- | --- | --- |
| CDHR3 | rs2528883 | GG | 83 | 86.12 |  | 72.06 |  |
|  |  | AA+AG | 91 | 81.69 | 0.146 | 72.73 | 0.746 |
|  |  | GG+AG | 155 | 82.96 |  | 71.62 |  |
|  |  | AA | 19 | 90.62 | 0.117 | 78.9 | **0.027** |
|  |  | GG | 83 | 86.12 |  | 72.06 |  |
|  |  | AG | 72 | 79.33 | **0.039** | 71.11 | 0.661 |
|  |  | GG | 83 | 86.12 |  | 72.06 |  |
|  |  | AA | 19 | 90.62 | 0.308 | 78.9 | **0.03** |
| CDHR3 | rs2526979 | GG | 53 | 80.48 |  | - | |
|  |  | AA+AG | 121 | 85.25 | 0.149 |  |  |
|  |  | GG+AG | 144 | 85.27 |  |  |  |
|  |  | AA | 30 | 76.75 | **0.034** |  |  |
|  |  | GG | 53 | 80.48 |  |  |  |
|  |  | AG | 91 | 88.06 | **0.019** |  |  |
|  |  | GG | 53 | 80.48 |  |  |  |
|  |  | AA | 30 | 76.75 | 0.47 |  |  |
| CDHR3 | rs34426483 | GG | 96 | 83.99 |  | - | |
|  |  | CC+GC | 78 | 83.57 | 0.89 |  |  |
|  |  | GG+GC | 162 | 84.76 |  |  |  |
|  |  | CC | 12 | 70.87 | **0.02** |  |  |
|  |  | GG | 96 | 83.99 |  |  |  |
|  |  | GC | 66 | 85.87 | 0.538 |  |  |
|  |  | GG | 96 | 83.99 |  |  |  |
|  |  | CC | 12 | 70.87 | **0.043** |  |  |
| CDHR3 | rs193806 | AA | 115 | - | | 71.86 |  |
|  |  | CC+CA | 59 |  |  | 73.49 | 0.457 |
|  |  | AA+CA | 169 |  |  | 72.9 |  |
|  |  | CC | 5 |  |  | 56.15 | **0.006** |
|  |  | AA | 115 |  |  | 71.86 |  |
|  |  | CA | 54 |  |  | 75.1 | 0.14 |
|  |  | AA | 115 |  |  | 71.86 |  |
|  |  | CC | 5 |  |  | 56.15 | **0.012** |
| CDHR3 | rs6967330 | GG | 152 | - | | 73.49 |  |
|  |  | AA+GA | 22 |  |  | 64.96 | **0.037** |
|  |  | GG+GA | 173 |  |  | 72.43 |  |
|  |  | AA | 1 |  |  | 70.39 | 0.882 |
|  |  | GG | 152 |  |  | 73.49 |  |
|  |  | GA | 21 |  |  | 64.71 | **0.04** |
|  |  | GG | 152 |  |  | 73.49 |  |
|  |  | AA | 1 |  |  | 70.39 | 0.807 |
| EMSY | rs2508740 | AA | 115 | 87.1 |  | 74.21 |  |
|  |  | GG+AG | 59 | 77.36 | **0.002** | 68.91 | **0.015** |
|  |  | AA+AG | 171 | 83.93 |  | 72.54 |  |
|  |  | GG | 3 | 76.7 | 0.538 | 65.24 | 0.358 |
|  |  | AA | 115 | 87.1 |  | 74.21 |  |
|  |  | AG | 56 | 77.4 | **0.003** | 69.11 | **0.021** |
|  |  | AA | 115 | 87.1 |  | 74.21 |  |
|  |  | GG | 3 | 76.7 | 0.361 | 65.24 | 0.241 |
| EMSY | rs7926009 | TT | 70 | 88.15 |  | 75.81 |  |
|  |  | CC+TC | 104 | 80.88 | **0.019** | 70.13 | **0.007** |
|  |  | TT+TC | 156 | 83.92 |  | 72.29 |  |
|  |  | CC | 18 | 82.76 | 0.816 | 73.51 | 0.719 |
|  |  | TT | 70 | 88.15 |  | 75.81 |  |
|  |  | TC | 86 | 80.48 | **0.021** | 69.42 | **0.003** |
|  |  | TT | 70 | 88.15 |  | 75.81 |  |
|  |  | CC | 18 | 82.76 | 0.279 | 73.51 | 0.492 |
| EMSY | rs2513525 | GG | 99 | 87.65 |  | 75.2 |  |
|  |  | TT+TG | 75 | 78.72 | **0.003** | 68.74 | **0.002** |
|  |  | GG+TG | 166 | 83.98 |  | 72.48 |  |
|  |  | TT | 8 | 80.11 | 0.596 | 71.1 | 0.781 |
|  |  | GG | 99 | 87.65 |  | 75.2 |  |
|  |  | TG | 67 | 78.55 | **0.004** | 68.46 | **0.002** |
|  |  | GG | 99 | 87.65 |  | 75.2 |  |
|  |  | TT | 8 | 80.11 | 0.284 | 71.1 | 0.375 |
| EMSY | rs4945087 | GG | 73 | 81.36 |  | 69.7 |  |
|  |  | AA+AG | 101 | 85.56 | 0.174 | 74.38 | **0.025** |
|  |  | GG+AG | 148 | 82.5 |  | 71.44 |  |
|  |  | AA | 26 | 91.19 | **0.041** | 77.96 | **0.024** |
|  |  | GG | 73 | 81.36 |  | 69.7 |  |
|  |  | AG | 75 | 83.61 | 0.51 | 73.14 | 0.122 |
|  |  | GG | 73 | 81.36 |  | 69.7 |  |
|  |  | AA | 26 | 91.19 | **0.035** | 77.96 | **0.014** |
| EMSY | rs2508746 | CC | 117 | 86.41 |  | 74.04 |  |
|  |  | TT+TC | 57 | 78.45 | **0.014** | 69.07 | **0.023** |
|  |  | CC+TC | 167 | 84.19 |  | 72.55 |  |
|  |  | TT | 7 | 74.46 | 0.21 | 69.07 | 0.508 |
|  |  | CC | 117 | 86.41 |  | 74.04 |  |
|  |  | TC | 50 | 79.01 | **0.027** | 69.07 | **0.031** |
|  |  | CC | 117 | 86.41 |  | 74.04 |  |
|  |  | TT | 7 | 74.46 | 0.118 | 69.07 | 0.326 |
| EMSY | rs1892953 | AA | 44 | 80.21 |  | 71.38 |  |
|  |  | GG+AG | 130 | 85.02 | 0.17 | 72.76 | 0.561 |
|  |  | AA+AG | 121 | 80.99 |  | 70.27 |  |
|  |  | GG | 53 | 90.23 | **0.001** | 77.31 | **0.002** |
|  |  | AA | 44 | 80.21 |  | 71.38 |  |
|  |  | AG | 77 | 81.43 | 0.766 | 69.64 | 0.505 |
|  |  | AA | 44 | 80.21 |  | 71.38 |  |
|  |  | GG | 53 | 90.23 | **0.016** | 77.31 | **0.038** |
| EMSY | rs2513513 | GG | 88 | 87.28 |  | 74.44 |  |
|  |  | AA+AG | 86 | 80.24 | **0.02** | 70.34 | **0.047** |
|  |  | GG+AG | 161 | 83.87 |  | 72.36 |  |
|  |  | AA | 13 | 82.98 | 0.879 | 73.09 | 0.854 |
|  |  | GG | 88 | 87.28 |  | 74.44 |  |
|  |  | AG | 73 | 79.75 | **0.02** | 69.86 | **0.03** |
|  |  | GG | 88 | 87.28 |  | 74.44 |  |
|  |  | AA | 13 | 82.98 | 0.449 | 73.09 | 0.731 |
| EMSY | rs2508755 | AA | 53 | 89.6 |  | 77.03 |  |
|  |  | GG+AG | 121 | 81.26 | **0.011** | 70.39 | **0.003** |
|  |  | AA+AG | 132 | 84.88 |  | 73.01 |  |
|  |  | GG | 42 | 80.42 | 0.211 | 70.53 | 0.305 |
|  |  | AA | 53 | 89.6 |  | 77.03 |  |
|  |  | AG | 79 | 81.71 | **0.019** | 70.32 | **0.004** |
|  |  | AA | 53 | 89.6 |  | 77.03 |  |
|  |  | GG | 42 | 80.42 | **0.023** | 70.53 | **0.024** |
| EMSY | rs1939469 | AA | 86 | - | | 74.1 |  |
|  |  | GG+GA | 88 |  |  | 70.76 | 0.106 |
|  |  | AA+GA | 162 |  |  | 72.11 |  |
|  |  | GG | 12 |  |  | 76.59 | 0.272 |
|  |  | AA | 86 |  |  | 74.1 |  |
|  |  | GA | 76 |  |  | 69.85 | **0.048** |
|  |  | AA | 86 |  |  | 74.1 |  |
|  |  | GG | 12 |  |  | 76.59 | 0.532 |
| EMSY | rs2155219 | TT | 54 | - | | 71.53 |  |
|  |  | GG+TG | 120 |  |  | 72.81 | 0.565 |
|  |  | TT+TG | 135 |  |  | 71.22 |  |
|  |  | GG | 39 |  |  | 76.54 | **0.031** |
|  |  | TT | 54 |  |  | 71.53 |  |
|  |  | TG | 81 |  |  | 71.02 | 0.835 |
|  |  | TT | 54 |  |  | 71.53 |  |
|  |  | GG | 39 |  |  | 76.54 | 0.104 |
| EMSY | rs12278256 | GG | 160 |  |  | 73.13 |  |
|  |  | TT+TG | 14 | - | | 64.2 | **0.018** |
|  |  | GG | 160 |  |  | 73.13 |  |
|  |  | TG | 14 |  |  | 64.2 | **0.018** |
| EMSY | rs3753051 | TT | 127 | 86.37 |  | - | |
|  |  | CC+TC | 47 | 76.86 | **0.005** |  |  |
|  |  | TT+TC | 173 | 83.89 |  |  |  |
|  |  | CC | 1 | 68.8 | 0.455 |  |  |
|  |  | TT | 127 | 86.37 |  |  |  |
|  |  | TC | 46 | 77.04 | **0.007** |  |  |
|  |  | TT | 127 | 86.37 |  |  |  |
|  |  | CC | 1 | 68.8 | 0.378 |  |  |
| EMSY | rs1044265 | AA | 59 | 83.27 |  | - | |
|  |  | GG+AG | 115 | 84.07 | 0.804 |  |  |
|  |  | AA+AG | 137 | 82.38 |  |  |  |
|  |  | GG | 37 | 89.07 | **0.029** |  |  |
|  |  | AA | 59 | 83.27 |  |  |  |
|  |  | AG | 78 | 81.7 | 0.668 |  |  |
|  |  | AA | 59 | 83.27 |  |  |  |
|  |  | GG | 37 | 89.07 | 0.118 |  |  |

Table S8 Functional prediction results by softwares RegulomeDB and HaploReg v4

| SNP | RegulomeDB score | Promoter histone marks | Enhancer histone marks | DNAse | Proteins bound | Motifs changed | Selected eQTL hits | site |
| --- | --- | --- | --- | --- | --- | --- | --- | --- |
| rs3847076 | 6 |  | 6 tissues |  |  | TCF4 |  | intronic |
| rs12278256 | 4 | 24 tissues |  | 51 tissues | 18 bound proteins | CTCF,TAL1,YY1 |  | upstream variant 2KB |
| rs144934374 | 2b | 24 tissues |  | 12 tissues |  | 10 altered motifs |  | upstream variant 2KB |
| rs2508746 | 5 |  | 12 tissues | 4 tissues |  | RXRA |  | 3'-flanking |
| rs1892953 | 4 |  | 11 tissues | 4 tissues |  | 4 altered motifs | 1 hit | 3'-flanking |

Note: For the RegulomeDB score, 2b means supporting data include TF binding + any motif + DNase Footprint + DNase peak; 4 means supporting data include TF binding + DNase peak; 5 means supporting data include TF binding or DNase peak; 6 represents other.

Figure S1 The volcano plots [of](https://en.wikipedia.org/wiki/Linkage_disequilibrium) significant SNPs.








Note: Fold change was minor SNP frequency in experimental group divided by minor SNP frequency in control group in dominant model (A) and recessive model (B). P value represented statistical significance of the relationship between all SNPs with asthma susceptibility in dominant model(A) and recessive model(B). Dotted line represented p=0.05.
